# Moving beyond attraction, compassion, and competence: evidence for compatibility as a distinct component of mate preferences

**DOI:** 10.3389/fpsyg.2026.1725609

**Published:** 2026-02-02

**Authors:** Peter Karl Jonason, Evita March

**Affiliations:** 1 Psychology Research Institute, VIZJA University, Warsaw, Poland; 2 School of Sciences, Psychology, and Sport, Federation University Australia, Ballarat, VIC, Australia

**Keywords:** dark triad, evolutionary psychology, life history theory, mate preferences, sex differences

## Abstract

**Introduction:**

Considerable research suggests there may be three primary qualities desired in romantic/sexual partners: physical attractiveness, interpersonal warmth, and social status. However, they might not capture the full range of needs served by what people seek in their partners; one omission may be compatibility.

**Methods:**

In one volunteer (*N* = 339, 26% male, Aged = 18–70 years, *M* = 30.36 years) and one Prolific (*N* = 309, 51% male, Aged = 18–79 years, *M* = 26.74 years) dataset, we assessed the relative importance of physicality (e.g., height, attractiveness), compassion (e.g., kindness, generosity), competence (e.g., social status, intelligence), and compatibility (e.g., interpersonal coordination) in mate preferences as a function of sex differences, context effects, and people's pace of life, mating strategies, and social strategies.

**Results:**

We replicated several established effects like physicality was valued in men strongly in the short-term context, and that more psychopathic and narcissistic people chose physicality more often in their long-term mates. Uniquely here, compatibility was more valued in the long-term context especially for women extending more parenting effort when considering short-term relationships and men who were less psychopathic when considering short-term relationships.

**Discussion:**

Importantly, our research begins to carve out a unique space for considering compatibility as a further higher-order trait worthy of consideration in mating research.

## Introduction

After 30 years of research on what people want in their long-term (i.e., romantic, marital) and short-term (i.e., casual, uncommitted) relationships, researchers agree that despite idiosyncratic preferences, people desire three main things in their partners that we will call physicality, competence, and compassion (Jonason and March, 2023; Jonason and Zagórska, 2025). Regardless of epistemological leanings, researchers agree that these preferences are a function—learned or evolved—of recurrent needs people have experienced (Buss and Schmitt, 1993; Eagly and Wood, 1999). In particular, seeking attractive (see Stephen and Luoto, 2022) partners serves needs of sexual arousal (and maybe species recognition), seeking partners who are compassionate affords them greater safety over the length of a courtship and relationship (Farrelly, 2013; Farrelly et al., 2016), and seeking partners who are competent can help one gain access to resources and solve day-to-day problems (Greitemeyer, 2007; Kasser and Sharma, 1999). These so-called Big Three traits have been repeatedly revealed in factor analytic research of (e.g., Fletcher et al., 1999, 2004) and proved important in experimental research (Li et al., 2002, 2013). However, factor analytic methods may suffer from being inductive and item-selection issues, and experimental studies may be subject to experimenter biases in the traits chosen. In two studies, we attempt to expand the Big Three of mate preferences to a Big Four, including compatibility, or the degree to which two people can coordinate a life together.

Biologically and sociologically, humans are rather unique in their mating behaviors. Unlike other mammals, human offspring require years—if not decades—of support to operate viably in the world. To successfully navigate this time, we assert that humans may have evolved active selection for compatible partners just like they have active selection for one's they find attractive (Luo, 2017; Luo and Klohnen, 2005). That is, a compatible partner satisfies the recurrent needs of interpersonal and intrafamilal coordination present in long-term pair bonded relationships. Compatible partners share values and have linguistic similarities (Daller and Ongun, 2024; Marchi et al., 2023), all of which may increase their ability to get along over long periods, especially when conflicts are more likely to occur over time and when dealing with serious intrarelational (e.g., reproductive timing) and extrarelational (e.g., raising housing costs) problems. Indeed, compatible partners have more relationship satisfaction (Baxter et al., 2022; but see Dyrenforth et al., 2010), which may be because they have less contentious arguments, fewer conflicts, and have an implicit understanding about important matters like religion (Furnham, 2009; Oda and Hayashi, 2020) and humor (Greengross and Miller, 2011). While most agree that people actively seek attractive, successful, and nice partners, there is much less work on the active selection (as opposed to convergence within couples; Csajbók et al., 2025; Luo, 2017) for compatible partners. This seems like a fundamental oversight given the unique nature of human romantic relationships, but also in lieu of the consensus that “similarity” is better than “opposites” in long-term, relationship stability and satisfaction. We attempt to add compatibility to the standard array of traits considered in mate preference research.

As previously noted “compatibility is theoretically crucial, but attempts to explain why certain perceived are compatibility with certain targets have revealed small effects” (Eastwick et al., 2023, p. 211). There are likely several reasons for this. First, researchers have often danced around the idea of compatibility assuming it plays a role in adjustment (Gottman, 2011), responsiveness (Reis and Gable, 2015), and intimacy (Sternberg, 1986) but have usually failed to consider it directly or as a feature of mate preferences focusing on relationship maintenance. Second, when considered, it is usually studied (1) in isolation and (2) not in relation to relationships that differ in level of commitment (Baxter et al., 2022; Luo, 2017) which can allow researchers to better understand its importance relative to other features but in men and women as a function of relationship commitment as would be noted as important by sexual strategies theory (Buss and Schmitt, 1993). Third, other research highlights the role of assortative or similarity in mate choice, preferences, or functioning which implies the importance of compatibility but cannot distinguish between whether similarity is a derived features from shared time or sought after as a quality in selection stages (Csajbók et al., 2025; Luo, 2017). Fourth, defining what it means to be compatible is elusive, and tends to be characterized by lower-order aspects and factor analytic methods (Marchi et al., 2023), not theoreticality-derived gestalt judgments based on deductive reasoning from a needs-based assessment. For example, people want partners who are like them in intelligence, level of education, and vocabularies all of which may hint at active selection (Daller and Ongun, 2024; Jonason and Antoon, 2019; Jonason et al., 2019) all of which are minor aspects that may serve needs for acquisition of resources and can be captured by the gestalt judgment of competence. And fifth, it is not uncommon for researchers (especially social psychologists) to fail to consider individual differences (see Jonason and Zagórska, 2025) in mating strategies (e.g., parenting effort), relationship psychology (e.g., attachment), and personality (e.g., narcissism). Therefore, in this replication with extension, we examine—in two studies—how much people prefer new partners who are compatible to them. We assessed the importance of compatibility relative to the Big Three traits of physicality, compassion, and competence. Further we integrate it into a sexual strategies model examining the interaction of sex and relationship context.

## The current study

We make several predictions for sex differences in preferences and expect them to be qualified by relationship context as per sexual strategies theory (Buss and Schmitt, 1993), which suggests that when men and women invest heavily in relationships they should differ less in what they want whereas when the minimum obligation differs or even conflicts, like in casual sex relationships, the sexes should differ more. First, we expect traits that have more utility in long-term relationships (i.e., compassion and compatibility) to be valued the most. Second, the values placed on each trait by men and women may be a function of asymmetries in investment in obligatory offspring and risk of within-relationship dangers like domestic violence. This may mean that women prioritize compassion more than men do whereas men prioritize physicality more than women do (Jonason and Antoon, 2019; Jonason et al., 2019). And third, while sex differences are likely to exist on their own, they will be calibrated to the context of the relationship, such that men and women may have similar mate preferences when they both are likely to invest heavily in the partnership and any potential offspring than when there is a greater asymmetry in that investment. Specifically, we expect women to have rather stable mate preferences across each context, whereas men will diverge from women in their mate preferences for short-term partners. Said another way, because women are exposed to more risk regardless of the context of the relationship, we expect their mate preferences to be relatively invariant across relationships contexts, but because men are not only at lower risks of things like domestic violence, they are not necessarily saddled with any offspring resulting from the sex, they may calibrate their mate preferences to the relationship context (Buss and Schmitt, 1993; Li et al., 2002; Li and Kenrick, 2006).

Beyond sex differences and relationship context effects, individual differences in psychosexual (e.g., sociosexuality, attachment) and psychosocial (e.g., psychopathy, care for family) strategies may relate to mate preferences (Afhami and Rafiee, 2020; Atari and Chegeni, 2017). We view these “strategies” through the lens of life history theory, as applied to personality traits (Figueredo et al., 2005; Giosan, 2006; Kruger, 2017), which suggests that there are within- and between-species differences in how individuals solve dilemmas created by the finite resources and time one has. Those with a “fast” or *r*-selected approach tend to have psychological systems that bias them toward immediate outcomes, limited investment, and risk. Those with a “slow” or *K*-selected approach tend to have psychological systems around altruism, family and kin care, and the avoidance of risk. While there is considerable research on mating psychology and personality psychology using a life history model, that research tends to be more concerned with sociosexuality, attachment, and the Dark Triad traits. Little of this research appears to focus on mate preferences but it seems reasonable that mate preferences could be a manifestation of life history strategies as captured in an array of individual differences.

To that end, we make several predictions. First, those motivated to find new sexual partners (i.e., high in mating effort) may be more interested in how attractive their partners are and less interested in how compassionate their partners are, especially in the short-term contexts when attractiveness is a priority and compassion is less important. Second, having a physically attractive partner is likely a priority for those interested in casual sex relationships because such relationships may be motivated by pleasure but, also, because this trait is the fastest—most apparent—to assess whereas other traits like compatibility may take weeks or months to assess. Third, those motivated to invest in and care for their family (i.e., high in parenting effort) should care more about qualities that will enable the long-term viability of romantic relationships (often irrespective of relationship context), such that they will desire compassionate, compatible, and competent partners more. Fourth, we conjecture that those who are avoidant in their attachment may have mate preferences that are expressions of their need to downregulate the stress they feel in relationships which means they may choose partners who are a little bit “toxic” (i.e., limited in compassion) and not a highly compatible because they need to have an escape route when feeling overwhelmed (i.e., they make emotionally safe choices that keep them single). And last, people who have a generally mutualistic approach to the world, as evidenced in a “slow” life history strategy, may be particularly attuned to traits that enable this mutualism, the most prominent here being compatibility.

While researchers—regardless of epistemological leanings or methodological styles—agree that the so-called Big Three are important features in mate selection, the features may not represent a complete list of the fundamental features or mate choice. Based on a needs assessment, we suggest that the need for a partner one can coordinate their relationship and life with (i.e., compatible with) may serve as an active mechanism—today more than ever—creating relationship homogamy and should be an important consideration in mate selection. To test this claim, we present two straight-forward studies, with brief assessment methods of mate preferences to understand sex differences and individual differences therein.

## Study 1: normative mate preferences

The most common method for understanding mate preferences is to ask people how much they like or desire particular qualities. However, most of this research draws on large banks of items. Instead, we focus only on four traits here to not only streamline the process, but to better examine multivariate processes and to reduce Type 1 error inflation. Our basic model combines the relative assessment of each trait in each context in two within-subjects' factors with the between-subjects factor of participants' sex. Then we turn to the correlations and explore their moderated nature by sex and context.

### Method

#### Participants and procedures

Data was collected from 339 participants (26% men) who were aged 18 to 70 years old (*M* = 30.36, *SD* = 10.15) and mostly heterosexual (72%), married (63%), and residing in Australia (38%). Although an initial 462 individuals accessed the questionnaire, 123 were excluded for reasons including not providing consent, not answering all the questions, failing the two attention checks, being under the age of consent, or not identifying as men or women. Participants were recruited via advertisements posted on social media and networking platforms (e.g., Reddit, Survey Circle, Survey Swap, and Facebook), which contained a link to the anonymous, voluntary online questionnaire. All participants provided informed consent before commencing the questionnaire and were debriefed and thanked upon completion. Participation took approximately 15 min. We attempted to collect a minimum sample size (*N*_one−*tailed*_ = 150) to detect the average effect size (*r* = 0.20) in social and personality psychology over the last 100 years (Richard et al., 2003). The hypotheses were not pre-registered. The data is available on the Open Science Foundation.^1^

#### Measures

Instead of using lengthy, multidimensional measures for each trait given the consensus around the Big Three traits, we crossed (and randomized) single items for each of the four mate preferences with each relationship context and asked participants whether each trait was important to them in each context (1 = *disagree strongly*; 5 = *agree strongly*). Prior to completing the questions, participants were provided with standardized definitions of each trait (see Appendix A) and were told that “a long-term relationship was defined as an intimate interpersonal relationship that involves commitment and that may be lifelong. Here the couple aims to build a loving and healthy relationship while creating a shared and balanced life together. A short-term relationship was defined as “a relationship which focuses on the immediate benefits and has no intention of developing into a long-term partnership. Some people may view this relationship as an experience that's more about fun and sex, and less about commitment.”

We measured individual differences in mating strategy and attachment with the 8-item Mating and Parenting Effort Scale (Kruger, 2017) and the 12-item self-report Experiences in Close Relationships Scale – Short Form (Wei et al., 2007). In both cases, participants reported their agreement (1 = *disagree strongly*; 5 = *agree strongly*) with items capturing mating effort (e.g., “Wear flashy expensive clothes”; α = 0.70), parenting effort (e.g., “Good at taking care of children”; α = 0.59), avoidant attachment (e.g., “I want to get close to my partner but, but I keep pulling back”; α = 0.78), and anxious attachment (e.g., “My desire to be very close sometimes scares people away”; α = 0.78). Items were summed to create three indexes.

### Results & discussion

We began with a 2 (sex) × 2 (context) × 4 (mating trait) mixed-model ANOVA with within-subjects on the last two factors (Table 1). We found a two-way interaction between trait and sex of the participant [*F*_(3, 1, 011)_ = 9.61, *p* < 0.001, η_*p*_^2^ = 0.03], suggesting that men valued a mate's physicality more than women, and valued compassion less than women. There were differences across participants' value of the mating traits [*F*_(3, 1, 011_) = 13.68, *p* < 0.001, $\eta_{p}^{2}$ = 0.04). Competence (*M* = 4.19, *SE* = 0.04) was rated as less important (*p* < 0.001) than physicality (*M* = 4.21, *SE* = 0.04), compassion (*M* = 4.42, *SE* = 0.04), and compatibility (*M* = 4.39, *SE* = 0.04), and physicality was less valued (*p* < 0.001) than compassion and compatibility, but this was qualified by an interaction with relationship context [*F*_(3, 1, 011)_ = 175.85, *p* < 0.001, $\eta_{p}^{2}$ = 0.34] suggesting that people valued all the traits more in the long-term (*M* = 4.56, *SE* = 0.03) than the short-term (*M* = 4.02, *SE* = 0.04) context in general but there was no differences between the value placed on compassion and compatibility (*p* < 0.25) in the long-term context and only a just-significant one in the short-term (*p* = 0.05), and differences between each trait across contexts were all significant (*p*s < 0.01) but the smallest was for physicality (0.23) compared to competence (0.84), compassion (0.73), and compatibility (0.89), suggesting that physical features preferences are more stable across context and the other three prioritized in the long-term context. People valued all the higher-order features more [*F*_(3, 1, 011)_ = 175.85, *p* < 0.001, $\eta_{p}^{2}$ = 0.34] in the long-term (*M* = 4.56, *SE* = 0.03) than the short-term (*M* = 4.02, *SE* = 0.04) context which is likely evidence of the tendency of people to be more interested in long-term than short-term relationships.

**Table 1:** Descriptive statistics and simple effects tests for short-term (STM) and long-term (LTM) mate preferences (Study 1).

	***M*** **(SD)**			***t***	***d***
	**Overall**	**STM**	**LTM**		
**Physicality**					
Men	4.33 (0.63)	4.45 (0.82)	4.20 (0.73)	3.07^**^	0.32
Women	4.08 (0.72)	4.19 (0.93)	3.98 (0.85)	2.61^*^	0.24
*t*	2.86^**^	2.38^*^	2.21^*^		
*d*	0.37	0.30	0.28		
**Competence**					
Men	4.11 (0.68)	3.66 (1.04)	4.56 (0.64)	−12.34^**^	−1.12
Women	4.26 (0.67)	3.88 (1.01)	4.65 (0.62)	−8.01^**^	−0.92
*t*	−1.86	−1.73	−1.20		
*d*	−0.22	−0.21	−0.14		
**Compassion**					
Men	4.32 (0.70)	3.91 (1.10)	4.73 (0.60)	−11.21^**^	−0.93
Women	4.52 (0.58)	4.21 (0.93)	4.84 (0.45)	−6.98^**^	−0.86
*t*	−2.71^*^	−2.46^*^	−1.84		
*d*	−0.31	−0.29	−0.21		
**Compatibility**					
Men	4.35 (0.62)	3.86 (1.10)	4.84 (0.43)	−13.14^**^	−1.17
Women	4.42 (0.59)	4.02 (0.94)	4.82 (0.51)	−8.23^**^	−1.06
*t*	−0.90	−1.28	0.40		
*d*	−0.12	−0.16	0.04		

Cohen's d for effect size.

^*^p < 0.05, ^**^p < 0.01.

Next, we correlated attachment and life history strategies with mate preferences (Table 2). Those higher in mating effort valued more physicality (especially men), and those higher in parenting effort valued more competence, compassion (especially men in the short-term context), and compatibility. Those high in parenting effort desired a partner who was competent (especially women in the short-term context), wanted a compassionate partner more (equally in both sexes), and wanted a compatible partner more (especially women in the short-term context). Avoidantly attached people wanted partners who were not all that compassionate (especially women overall and men in the long-term) and desired a long-term partner who was less compatible (especially in the women). And last, anxious attachment was nearly uncorrelated with mate preferences except in men's preference for a less compatible long-term partner.

**Table 2:** Correlations between mate preferences and individual differences by long-term (LTM) and short-term (STM) contexts overall and in men (*n* = 88) and women (*n* = 251) in Study 1.

	**Physicality**			**Competence**			**Compassion**			**Compatibility**		
	**Overall**	**LTM**	**STM**	**Overall**	**LTM**	**STM**	**Overall**	**LTM**	**STM**	**Overall**	**LTM**	**STM**
**Mating effort**	0.12^*^	0.04	0.15^**^	0.06	0.09	0.03	−0.14	−0.07	−0.14^*^	−0.06	0.01	−0.08
Men	0.36^**^	0.23^*^	0.35^**^	0.09	0.07	0.07	−0.23^*^	−0.22^*^	−0.17	−0.12	−0.11	−0.10
Women	−0.01	−0.06	0.04	0.09	0.12	0.05	−0.05	0.04	−0.08	−0.02	0.05	−0.05
**Parenting Effort**	−0.01	−0.01	< 0.01	0.16^**^	0.07	0.18^**^	0.32^**^	0.25^**^	0.27^**^	0.20^**^	0.11	0.18^**^
Men	−0.18	−0.02	−0.25^*^	0.07	−0.04	0.12	0.30^**^	0.26^*^	0.25^*^	0.20	0.14	0.17
Women	0.08	0.02	0.10	0.19^**^	0.11	0.18^**^	0.30^**^	0.23^**^	0.26^**^	0.19^**^	0.10	0.18^**^
**Avoidant Attachment**	−0.03	0.02	−0.06	−0.07	−0.10	−0.03	−0.16^**^	−0.19^**^	−0.10	−0.07	−0.13^*^	−0.02
Men	−0.02	−0.06	0.02	0.06	−0.03	0.10	−0.09	−0.25^*^	0.02	−0.16	−0.11	−0.14
Women	−0.04	0.04	−0.09	−0.11	−0.13^*^	−0.07	−0.18^**^	−0.17^**^	−0.14^*^	−0.04	−0.14^*^	0.03
**Anxious Attachment**	−0.02	−0.02	−0.02	−0.06	−0.08	−0.04	< 0.01	0.04	−0.02	−0.03	−0.04	−0.01
Men	−0.14	−0.15	−0.09	0.02	−0.13	0.05	0.01	0.06	−0.02	−0.15	−0.23^*^	−0.09
Women	0.02	0.03	< 0.01	−0.08	−0.07	−0.07	−0.02	0.03	−0.03	0.02	0.01	0.02

^*^p < 0.05, ^**^p < 0.01.

## Study 2: choosing between traits

The assessment of normative questions serves to assess ideal or expressed preferences. However, this approach—albeit common—may be subject to social desirability effects along with limited ecological validity. Therefore, instead, we adopt another method, one that will capture expressed preferences such that we use a behavioroid task to see how often people choose each trait relative to the others. We adopt the analytical approach as above, with a mixed model ANOVA, correlational analyses, and the exploration of moderation by sex and context.

### Method

#### Participants and procedures

Participants were 309 Prolific workers from Australia (51% men) aged 18 to 79 (*M* = 26.74, *SD* = 9.00) who were predominantly heterosexual (76%), had a university degree (65%), and claimed to be “native” in English (79%). While 386 people started the survey, we removed 26 for failing the attention check and 51 for failing to provide complete data or withdrawing from the survey. Participants were first informed about the study and the voluntary nature of their participation. If they agreed to participate, they provided tick-box consent. Participation took approximately 10 min, and upon completion they were thanked and debriefed. The hypotheses were not pre-registered. The data is available at the same on-line location as Study 1.

#### Measures

Mate preferences were assessed with a dilemma task (Jonason and Zagórska, 2025) in which participant were asked to choose between all pairs of physicality, compatibility, compassion, and competence for a long-term and short-term mate, defined like in Study 1. For instance, participants were asked to choose which they wanted more for a long-term partner, compatibility or competence. We summed the times each trait was chosen within each context.

We measured pace of life with the 26-item High *K* Strategy Scale (Giosan, 2006), mating strategies with the 21-item Multidimensional Sociosexual Orientation Inventory (Jackson and Kirkpatrick, 2007)^2^, and personality with the 12-item Dirty Dozen measure of the Dark Triad traits (Jonason and Webster, 2010). In all three cases participants reported their agreement (1 = *strongly disagree*; 5 = *strongly agree*) with statements measuring *fast*-to-*slow* life history strategies (e.g., “I am able to provide a decent quality of life for myself and my family”; α = 0.84)^3^, interest in short-term relationships (e.g., I can imagine myself enjoying a brief sexual encounter with someone I find very attractive”; α = 0.88), interest in long-term relationships (e.g., “I would like to have a romantic relationship that lasts forever”; α = 0.79), Machiavellianism (e.g., “I tend to manipulate others to get my way”; α = 0.74), narcissism (e.g., “I tend to seek prestige or status”; α = 0.76), and psychopathy (e.g., “I tend to be cynical”; α = 0.63). The items were averaged for each scale.

### Results & discussion

We tested a mixed model ANOVA with a 2 (participant's sex) × 2 (mating context) × 4 (mating trait) design (see Table 3). We detected a main effect of mating trait [*F*_(3, 307)_ = 131.87, *p* < 0.001, $\eta_{p}^{2}$= 0.30]; overall, participants chose compatibility more than compassion (*p* < 0.001), compassion more than physicality (*p* < 0.05), and physicality more than competence (*p* < 0.001). We detected an interaction between sex and mating trait [*F*(3, 307) = 9.62, *p* < 0.001, $\eta_{p}^{2}$= 0.03], suggesting that men chose physicality more than women (*p* < 0.001). Men cared about competence the least, choosing it less than physicality (*p* < 0.01), compassion (*p* < 0.05), and compatibility (*p* < 0.001), instead choosing compatibility most of all, prioritizing it over physicality (*p* < 0.001), compassion (*p* < 0.01), and competence (*p* < 0.001). Similarly, women chose compatibility more than physicality (*p* < 0.001), compassion (*p* < 0.001), and competence (*p* < 0.001). Additionally, women chose compassion less than competence (*p* < 0.001) and physicality (*p* < 0.001), they chose physicality less than compatibility (*p* < 0.001), and chose competence less than compatibility (*p* < 0.001).

**Table 3:** Descriptive statistics and simple effects for how many times people chose each trait (Study 2).

	**Mean (** ***SD*** **)**			***t***	***d***
	**Overall**	**Men**	**Women**		
**Physicality**					
Long-term	0.59 (0.72)	0.73 (0.76)	0.44 (0.65)	3.62^**^	0.41
Short-term	2.13 (0.94)	2.40 (0.84)	1.85 (0.96)	5.29^**^	0.61
*t*	−25.27^**^	−19.90^**^	−16.05^**^		
*d*	−1.44	−1.58	−1.31		
**Compassion**					
Long-term	1.81 (0.83)	1.82 (0.85)	1.80 (0.81)	0.16	0.02
Short-term	1.25 (0.95)	1.18 (0.91)	1.32 (0.98)	−1.25	−0.15
*t*	9.05^**^	7.07^**^	5.69^**^		
*d*	0.52	0.56	0.46		
**Competence**					
Long-term	1.05 (0.87)	0.94 (0.90)	1.17 (0.83)	−2.32^*^	−0.27
Short-term	0.81 (0.88)	0.72 (0.82)	0.89 (0.93)	−1.74^*^	−0.19
*t*	4.21^**^	2.78^**^	3.15^**^		
*d*	0.24	0.22	0.26		
**Compatibility**					
Long-term	2.54 (0.70)	2.52 (0.77)	2.56 (0.61)	−0.56	−0.06
Short-term	1.77 (1.02)	1.70 (0.98)	1.85 (1.05)	−1.37	−0.15
*t*	12.89^**^	10.54^**^	7.84^**^		
*d*	0.73	0.84	0.64		

d is Cohen's d.

^*^p < 0.05, ^**^p < 0.01.

We detected an interaction between context and mating trait [*F*(3, 307) = 233.42, *p* < 0.001, $\eta_{p}^{2}$= 0.43], with participants choosing compassion (*p* < 0.001), compatibility (*p* < 0.001), and competence (*p* < 0.001) more in the long-term context than in the short-term context. On the other hand, participants chose physicality more in the short-term context than in the long-term context (*p* < 0.001). In the long-term context, participants chose compatibility more than compassion (*p* < 0.001), competence more than physicality (*p* < 0.001), and compassion more than competence (*p* < 0.001). In the short-term context, participants chose physicality more than compatibility (*p* < 0.001) and compassion more than competence (*p* < 0.001).

Next, we correlated the personality traits with how much each mating trait was chosen (Table 4). Those who chose physicality more in the long-term context were more psychopathic, more narcissistic (especially men), had a short-term mating orientation (especially women), and were men who were disinterested in long-term relationships. Those who chose physicality more in the short-term context were more Machiavellian men, those interested in short-term and long-term relationships. Those who chose compassion more were less psychopathic, and Machiavellian (especially women) and women interested in long-term relationships. Those who chose compassion for their short-term mates were narcissistic men and non-Machiavellian women. Those who chose competence in their long-term partners were more likely to be fast life history, psychopathic, short-term oriented, but less narcissistic men. Those who chose competences in their short-term partners were more psychopathic (especially women) and were men who were disinterested in short-term relationships and women were disinterested in long-term relationships. Those who chose compatibility for their long-term partners were more likely to be slow life history strategists (especially men) and to be men who were less psychopathic. And last, those who chose compatibility in their short-term partners were less psychopathic and Machiavellian men.

**Table 4:** Correlations between construct choices and individual differences by relationship context and participant's sex (Study 2).

	**Physicality**		**Compassion**		**Competence**		**Compatibility**	
	**LTM**	**STM**	**LTM**	**STM**	**LTM**	**STM**	**LTM**	**STM**
**Life history strategy**	0.02	−0.03	−0.06	−0.06	−0.10	0.02	0.20^**^	0.07
Men	0.01	0.04	−0.11	−0.06	−0.16^*^	−0.03	0.30^**^	0.05
Women	0.08	−0.04	< 0.01	−0.07	−0.06	0.06	0.04	0.09
**Psychopathy**	0.12^*^	0.08	−0.11^*^	−0.06	0.09	0.11^*^	−0.08	−0.11^*^
Men	0.03	0.07	< 0.01	< 0.01	0.14^*^	0.09	−0.20^*^	−0.13^*^
Women	0.13	−0.06	−0.27^**^	−0.09	0.11	0.20^**^	0.12	−0.05
**Narcissism**	0.12^*^	0.07	−0.01	< 0.01	−0.05	−0.04	−0.05	−0.04
Men	0.15^*^	0.03	0.04	0.13^*^	−0.14^*^	−0.09	−0.03	−0.07
Women	0.10	0.13	0.06	−0.12	0.04	−0.01	−0.07	0.01
**Machiavellianism**	−0.04	0.06	−0.13^*^	−0.11	0.10	0.05	< -0.01	< -0.01
Men	0.02	0.16^*^	−0.06	−0.04	0.10	0.04	−0.07	−0.14^*^
Women	0.03	−0.06	0.22^**^	−0.17^*^	0.13	0.07	−0.08	0.13
**Short-term Orientation**	0.17^**^	0.17^**^	−0.02	−0.04	−0.09	−0.06	< -0.01	0.07
Men	0.10	0.07	< 0.01	0.05	−0.03	−0.16^*^	−0.06	0.03
Women	0.17^*^	0.13	−0.04	−0.09	−0.10	0.07	0.09	−0.02
**Long-term Orientation**	−0.03	0.12^*^	0.06	< 0.01	< -0.01	−0.10	−0.05	−0.02
Men	−0.17^*^	0.02	−0.02	−0.01	0.20^**^	0.05	−0.04	−0.05
Women	−0.02	0.03	0.14^*^	0.06	−0.11	−0.17^*^	−0.05	0.04

LTM, long-term mating context; STM, short-term mating context. ^*^p < 0.05, ^**^p < 0.01.

## General discussion

Based on rigorous psychometric (Fletcher et al., 1999, 2004) and experimental (Li et al., 2002, 2013) research, there is a consensus that while there might be idiosyncratic manifestations of mate preferences, there are at least three primary dimensions that influence people's decisions, we call these preferences physicality, compassion, and competence. Each reflects underlying needs that people are likely to have had over selection for mating psychologies and continue to exert pressures across sociological and developmental periods. For instance, the needs created for sexual arousal can be satisfied by finding a physically attractive partner, the need for safety within a relationship can be solved by finding a partner who is “nice” (i.e., willingness to invest), and the needs created by existential threats like finding food can be solved by finding a partner who is intelligent and capable (i.e., ability to invest). We contend here that these may be insufficient given the uniquely lengthy and coordinated social lives of those who pair up and the extreme helplessness of human offspring relative to other species, including closely related mammals like higher order primates, along with the reliance on factor analytic approaches to derive the three factors. To address this, our studies propose an additional variable to consider in addition to the Big Three mate preferences: compatibility.

Unlike the other three preferences, compatibility has received limited support and only recently at that (Baxter et al., 2022; Daller and Ongun, 2024; Marchi et al., 2023; Luo, 2017; Luo and Klohnen, 2005). Our studies focused on the ideas that (1) people have active selection mechanism for compatibility (a.k.a., homogamy, assortative mating), (2) these preferences should be studied in relation to other mate preferences given the multidimensional nature of mate choice, and (3) there are individual differences in who values compatibility based on their psychosocial strategies, sex, and the level of investment demanded in different relationships. Therefore, our studies are replications with extension relying on two methods of assessment of mate preferences whereby we study a finite list of traits desired in partners, examine them in relation to the interaction of sex and context ala sexual strategies theory, and we explore individual differences in those mate preferences with traits like narcissism, parenting effort, and attachment ala life history theory.

We found several sex differences consistent with previous work, but several things stand out. First, differences in preferences for all four traits were usually larger within-sex than between, especially for men, which aligns with the idea that the sexual strategies theory is more about context than sex differences and that men's mate preferences are more malleable to the level of investment than women's (Jonason and Antoon, 2019; Jonason et al., 2019). Second, no sex differences emerged for competence, appearing to violate the idea that women need a partner who can provide more than men do but, given the W.E.I.R.D. (i.e., Western, Educated, Industrialized, Rich, and Democratic) nature of our samples, we find this interpretation less likely than sampling error. Nevertheless, competence may be more than income or resources (Jonason and Thomas, 2022) and, thus both partners should want a partner who is competent. Indeed, one reason we prefer these larger terms like competence rather than income is that we do not think there was active selection for income so much as income was and is a cue to competence. Third, and otherwise, although no difference was observed for long-term partners, women wanted a short-term partner who was compassionate and compatible more than men did, suggesting that (1) either men will discount safety concerns for a relationship that is unlikely to last but women are not similarly willing or (2) women maintain their preference for nice and successful men because of the benefits they can get from that kind of man. Fourth, men and women agreed, that regardless of context, compatibility was important in their mating decisions as much (Study 1) if not more (Study 2) than compassion. Whether it be in sexual relationships or committed ones, compatibility is likely to enable sexual and relationship satisfaction along with the process of getting to know one another over the course of courtship no matter if it is over an hour or over a year. And fifth, the ipsative method from Study 2 appears to either amplify (i.e., measurement error) or reveal (i.e., reduces social desirability) sex differences especially suggesting that it might be a useful method (Jonason and Zagórska, 2025). If the method does create more variability, it might be especially useful to assess the correlations with other individual differences because other methods like the budget allocation may suffer too much from range restriction, especially in the low budget conditions (Li et al., 2002, 2013).

Sex differences alone are underwhelming because there is within-sex variance as well. To address this, we examined several psychosocial strategies in terms of mating effort (i.e., a general way of life), attachment (i.e., a style of relationships), and psychopathy (i.e., a style of social interaction) in relation to mate preferences overall and in each relationship context.^4^ We focus our discussion, however, on compatibility only. We test the tentative assertion that mate preferences might not merely be correlated with these “strategies” but instead, serve needs created by their life history strategies and each of these constructs captures different shades of fast and slow life history strategies.

In Study 1, those who place more effort into parenting/kin care valued compatibility more, an effect that was trivially (*p* < 0.08) stronger in the short-term context. For those who value long-term mutualistic relationships, it seems logical for them to emphasize a trait that is especially important in that same context. In fact, the effects of localization in women further underlie this point given that women are more likely to emphasize parenting and family motivations in their lives. In addition, men who were anxiously attached and women who were avoidantly attached appear to devalue compatibility in the long-term context which, if trustworthy, may be a way of regulating commitment; avoidant women seeking long-term partners who are less compatible to minimize engulfment risks and anxious men seeking long-term partners who are less compatible because it creates the “chase” that their nervous system needs.

In Study 2, compatibility preferences were stronger among slow life history strategist men in the long-term context suggesting an alignment of trait, context, and life history strategy that would lead to a slow, but effective, reproductive strategy. In contrast, psychopathic men chose compatibility less regardless of context suggesting that psychopathic men may not only be calibrated to not seek partners with whom they can build long-term relationships with, they have a blanket aversion toward incompatibility which may reflect a preferred antagonistic interpersonal life. Indeed, Machiavellian men chose compatibility less as well, but only in the short-term context which may be an adaptive calibration for men in that it is context specific; being compatible with a long-term partner may fit Machiavellian people's long-term goals such a trait in their short-term partners has little utility. While the various personality traits we examined provided limited and even weak evidence for their role in accounting for this and other mate preferences, they do appear to align with prior research well, like physical traits being chosen more for those interested in short-term mating, those interested in parenting were quite concerned with their partner's compassion (Robey and Sori, 2012), and avoidant women not wanting relationships that are too intimate (i.e., less compassion). It must be noted however, that the tests for moderation by sex were rather weak, so we comparisons men and women in an exploratory spirit only.^5^

## Limitations & conclusions

While we adopted two face-valid measurement approaches to study the importance people place on compatibility in their relationships overall and in relation to the Big Four traits, along with who—in terms of personality writ large—desires each, our study has several limitations. First, our reliance on single-item self-reported assessments may give traditional psychometricians pause (Haeffel and Howard, 2010), even though we used definitions to standardize responses which, we think, serve to augment the shortcomings of single-items. Moreover, our second method was based on choices capturing revealed preferences not self-reports with normative assessments to capture expressed preferences which is the most common method of assessment of traits, preferences, attitudes, and more. Second, we relied on a relatively limited set of personality traits of the participants, all of which are (1) brief in nature and (2) cannot be reduced to aspects of each trait. Third, our research may be more informative to heterosexual than homosexual couples if we assume that sexual orientation generates unique mate selection processes in each group. Fourth, by focusing on short-term and long-term relationships only, we may be creating a false dichotomy and omitting important nuances we could learn from hybrid relationships like polyamory or friends-with-benefits. Fourth, while we did not use the Mini-*K* (Figueredo et al., 2005), which is particularly dubious in terms of capturing life history strategies (Gruijters and Fleuren, 2018), the two measures we used may nevertheless be limited, not necessarily in psychometrics, but in conceptualization (Zietsch and Sidari, 2020; but see Woodley of Menie et al., 2021). Fifth, mate preferences should be calibrated to living conditions, but our research was on individual differences more than contextual effects. The short-term v. long-term distinction is less context and more a proxy for level of investment. And sixth, we cannot say whether four traits are *the* necessary and sufficient traits to capture the full range of qualities people seek in their partners. Indeed, we can see cause for at least one more—character—as another important feature that enables the long-term coordination of relationships (Gottman, 2011; Sternberg, 1986). Future research should address these matters (e.g., cross-national studies, broader personality traits and individual difference assessment), but until then, we think our results triangulate within our studies and extend what is known about mate preferences.

What do people want in their romantic and sexual partners? We contend that this is best answered by asking what they need. People need to feel aroused; people need to feel safe in their relationships; people need to feel safe in the world; and people need to feel heard. We contend the first of these are well studied in the so-called Big Three traits and we add here that active preferences—a mechanism creating homogamy—for compatible partners may serve the last. Partnering with those who are more compatible will enable the long-term coordination and problem-solving characterizing monogamous mating systems around the world which are especially strong in *Homo sapiens*. In this study, we replicated and extended the Big Three model to a Big Four model by studying the relative value placed in compatibility in concert with physicality, compassion, and competence preferences in two online samples, with two straight-forward measures of mate preferences. We focused on sex and context effects along with induvial differences in romantic, life, and social strategies.

We expect the integration of compatibility with the Big Three traits is likely to have basic and applied implications. First, by having a short, standard list of mate preference characteristics, researchers from various disciplines can focus on the same, finite list in correlational, cross-national, and experimental research thereby avoiding the jingle-jangle fallacy, reducing noise in the field, and focusing our attention on deeper needs rather than some of the idiosyncratic minutia that characterizes much of this field. Second, if this—or some subsequent-yet-similarly-brief—list of needs, as opposed to wants, may allow therapists and dating coaches to better advise their clients in ways to maximize their relationship satisfaction by not just determining what their needs are and their origins, but also help the clients distinguish between the essential and simply desirable features they may want. Taken together, we think such an approach might be the beginning of large scale, coordinated, projects that will move research on this important aspect of human nature from petty squabbles and grand(er) questions.

## Data Availability

The datasets presented in this study can be found in online repositories. The names of the repository/repositories and accession number(s) can be found below: https://osf.io/bjuys/.

## Footnotes

1 https://osf.io/bjuys.

2 We excluded the previous sexual experience factor.

3 For the High-*K* scale, because most participants (73%) were not married or did not have a partner, we omitted these items in our calculations.

4 Given the within-subjects nature of the relationship context distinction, we felt it was trustworthy to make such comparisons.

5 We suspect better science in this area requires a doubling of sample sizes to best test moderation if we assume 250 people are needed for correlations to stabilize.

## Appendixes

**Appendix A**. Definitions of super-categories of mate preferences given to participants.

*Physicality* can be defined as the degree to which the physical traits of an individual are perceived as pleasing or beautiful. It has to do with the way individuals look, their height/weight, and facial appearance/complexion.

*Compassion* can be defined as a feeling of sympathy with another's feelings, usually involving a desire to help or comfort that person. It refers to the tendency of a person to be nice, generous, kind, to care for others, to listen, and offer help.

*Competence* can be defined as the ability to do something well or effectively in a certain field. It has to do with an individual's social and/or professional status, income, ambitiousness, motivation, and success.

*Compatibility* can be defined as the degree to which you perceive someone as being like you in important ways. It refers to the perception of sharing similar interests, values, attitudes, opinions, and moral priorities.
